# Role of c-Src activity in the regulation of gastric cancer cell migration

**DOI:** 10.3892/or.2014.3188

**Published:** 2014-05-15

**Authors:** YUN YANG, ZHI-GANG BAI, JIE YIN, GUO-CONG WU, ZHONG-TAO ZHANG

**Affiliations:** Department of General Surgery, Beijing Friendship Hospital, Capital Medical University, Xuanwu, Beijing 100050, P.R. China

**Keywords:** c-Src, gastric cancer, furin, MT1-MMP, VEGF-C, cell migration

## Abstract

Gastric cancer is associated with increased migration and invasion. In the present study, we explored the role of c-Src in gastric cancer cell migration and invasion. BGC-823 gastric cancer cells were used to investigate migration following treatment of these cells with the c-Src inhibitors, PP2 and SU6656. Migration and invasion were analyzed by wound healing and Transwell assays. Western blot analysis was used to detect the expression of MT1-MMP and VEGF-C, while the activity of MMP2 and MMP9 was monitored with gelatin zymography assay. Immunoprecipitation was used to detect interactions among furin, pro-MT1-MMP and pro-VEGF-C. MT1-MMP and VEGF-C expression levels were inhibited by PP2 and SU6656 treatment, in accordance with decreased c-Src activity. Similarly, the zymography assay demonstrated that the activity of MMP2 and MMP9 was decreased following PP2 or SU6656 treatment. Blockade of c-Src also inhibited the invasive and migratory capacity of BGC-823 cells. Notably, c-Src interacted with furin *in vivo*, while interactions between furin and its substrates, pro-MT1-MMP and pro-VEGF-C, were decreased by c-Src inhibitors. In conclusion, the interaction among furin and pro-MT1-MMP or pro-VEGF-C or other tumor-associated precursor enzymes can be regulated by c-Src activity, thus reducing or changing the expression of these enzymes in order to reduce the development of gastric cancer, invasion and metastasis.

## Introduction

Gastric cancer is the leading cause of cancer-related mortality worldwide and its incidence continues to rise in both developed and developing countries (1). Population-based studies have shown that the incidence rate approximates the death rate, which is >730,000 annually. Hence, most patients who develop gastric cancer will succumb to this disease (2). Gastric cancer itself is highly malignant and exhibits an inherited predisposition to infiltrate and metastasize. At present, the mechanisms underlying gastric cancer initiation, progression and metastasis are not fully understood (3).

It is well known that cellular proteins must be cleaved by protein convertases before maturation. Furin is the best characterized representative of the mammalian subtilisin-like family of proprotein convertases. The propeptide-furin complex leaves the endoplasmic reticulum (ER) and enters the trans-Golgi network (TGN) for its second activational cleavage (4). This step results in furin, which can process substrates in multiple compartments in the TGN/endosomal system (5). Among the known furin substrates are precursors of hormones, neuropeptides, growth factors, adhesion molecules, receptors, surface proteins, viral glycoproteins and bacterial toxins (6–8). Two cellular migration and invasion proteins, pro-MT1-MMP and vascular endothelial growth factor (VEGF), must also be cleaved by furin for their activation (9).

Due to its important role in cleavage-mediated protein activation, furin is considered a potential prognostic, or even therapeutic, factor for tumorigenesis. Therefore, many medical center doctors, including us, have become interested in the mechanisms by which furin may be involved in so many important biochemical, clinical and therapeutic functions in tumor research (10).

As a member of the Src family of non-receptor tyrosine kinases, c-Src is often upregulated in a variety of human tumors, including gastric cancer (11–13). c-Src functions as a critical link between multiple signaling pathways that regulate proliferation, invasion, survival, metastasis and angiogenesis (14,15). Molecular-targeted therapy of c-Src has thus emerged as a promising treatment of gastric cancer. For example, one potential target is the Src family kinase (SFK).

However, although c-Src and furin have both been found to be upregulated in human cancer (16,17), whether the ubiquitously expressed c-Src participates in the interactions between furin and its substrates remains unknown.

In the present study, we detected the protein levels and the interactions between furin and its substrates after either stimulation with epidermal growth factor receptor (EGFR) ligands or treatment with c-Src inhibitors in BGC-823 cells. In the present study, we demonstrated that activation of furin is c-Src dependent in gastric cancer cells and thus targeting the furin-c-Src interface could be a promising strategy against gastric cancer progression and metastasis.

## Materials and methods

### Cell culture and experimental reagents

The gastric cancer cell line BGC-823 was cultured in RPMI-1640 (Invitrogen, USA) supplemented with 10% fetal bovine serum (FBS), 100 U/ml penicillin and 100 μg/ml streptomycin. All cells were cultured in a 5% CO_2_ humidified atmosphere at 37°C. The EGFR agonist PDGF-BB (20 ng/ml, R&D, USA) or c-Src inhibitors, PP2 or SU6656 (10 μM each), were added to cells cultured in serum-free medium.

Primary antibodies against pSrc (Y416), furin, MT1-MMP, VEGF-C and β-actin were purchased from Santa Cruz Biotechnology (Santa Cruz, CA, USA). The gelatin zymography kit was from Millipore (USA) while 4-amino-5-(4-chlorophenyl)-7-(t-butyl) pyrazolo [3,4-d] pyrimidine (PP2) and PDGF-BB were purchased from Enzo Life Sciences International (USA). SU6656 was purchased from Sigma (USA).

### Gelatin zymography

Levels of the active and latent forms of MMP2 and MMP9 were analyzed by gelatin zymography as described in the manufacturer’s instructions. Briefly, BGC-823 cells were washed with ice-cold PBS and lysed with RIPA buffer for 30 min on ice. Lysates were then cleared by centrifugation at 12,000 × g for 20 min at 4°C. The supernatant was aliquoted and protein content was determined using the BCA method (Pierce). Equal amounts of protein were separated by gel electrophoresis. The gel was subsequently washed and incubated at 37°C for 24 h, then stained with Coomassie brilliant blue R250. Bands were examined after the gel was destained by Coomassie Blue Staining Destaining Solution.

### Wound healing assay

BGC-823 cells were grown to confluence in 24-well plates. The monolayer was then artificially wounded using a sterile 200-μl pipette tip. Cell debris was removed by washing the monolayer with PBS. The cells were then incubated with c-Src inhibitors, PP2 or SU6656, at a dose of 10 μM. Wound closure was monitored by photographing cell migration into the wound at various time points at the same spot with an inverted microscope equipped with a digital camera. The extent of healing was defined as the ratio of the difference between the original and the remaining wound areas compared with the original wound area.

### Transwell invasion assay

Matrigel invasion chambers were hydrated for 4 h before starting the invasion assay. Log-phase cells (4×10^4^) were then plated in 200 μl RPMI-1640 containing 10% FBS in the upper chambers of the Transwell. The lower chambers were filled with 500 μl RPMI-1640 containing 10% FBS. After incubation for 2 h, the cells were treated with 10 μM of either PP2 or SU6656 for 24 h. The cells were then allowed to migrate for 10 h in the cell culture incubator. Then, the cells were fixed for 15 min at room temperature by replacing the culture medium in the bottom and top chambers with 4% formaldehyde dissolved in PBS. The cells that remained on the bottom of the chamber were stained with 0.1% crystal violet and photographed under an optical microscope. The cell number was counted in 12 different fields of view. Data were averaged from three parallel experiments, which were normalized to those of the non-treated control.

### Western blot analysis

Cells were lysed in RIPA buffer [50 mM Tris (pH 7.4), 150 mM NaCl, 1% Triton X-100, 0.1% SDS, 1% sodium deoxycholate, 5 mM EDTA, 100 mM NaF and 1 mM Na_3_VO_4_] containing protease inhibitor cocktail for 30 min at 4°C. Cell lysates were then cleared by centrifugation at 4°C at 16,000 × g for 30 min. The protein concentrations were determined by the BCA (bicinchoninic acid) method (Pierce, USA) and then equal protein amounts of cell lysates were fractionated by electrophoresis in SDS-PAGE. Gels of 10% were used for the analysis of furin and c-Src, while 12% gels were used to analyze MT1-MMP and VEGF-C. Following electrophoresis, proteins were electroblotted onto polyvinylidene fluoride (PVDF) membranes using a wet transblot system (Bio-Rad, Hercules, CA, USA). Membranes were then blocked with 10% bovine serum albumin (BSA) or 5% non-fat dry milk for 1 h at room temperature. Membranes were next incubated overnight at 4°C with antibodies against pSrc (Y416), furin, MT1-MMP, VEGF-C and β-actin, all diluted 1:1,000 in phosphate-buffered saline with Tween-20 (PBST). After several washes in PBST, the membranes were incubated for 1 h with horseradish peroxidase-conjugated goat anti-rabbit or anti-mouse secondary antibodies, each diluted 1:5,000 in PBST. Membranes were washed as before and the immunoreactive bands were processed using the Super Signal West Pico chemiluminescent substrate (Pierce, USA), followed by exposure to the Fujifilm LAS3000 Imager (Fuji, Japan). Densitometric analysis was performed with the Image J densitometer and Excel software.

### Co-immunoprecipitation (co-IP)

BGC-823 cells were washed twice with ice-cold PBS, lysed in 1 ml RIPA buffer for 30 min on ice and clarified by centrifugation at 4°C at 10,000 × g. The supernatant was then collected and subjected to IP. Briefly, each cell lysate (500 μg) was incubated with 2 μg of the appropriate antibody (anti-c-Src or anti-furin) overnight at 4°C. Protein G (50 μl) was then added and the mixture was incubated at 4°C for 2 h with gentle agitation. The pellet was retrieved by centrifugation and washed three times with RIPA buffer. It was then boiled with 50 μl 2X loading buffer (Tris pH 6.8, 0.1% SDS, 10% glycerol and 0.025% bromophenol blue, 20 mM DTT) for 5 min prior to gel loading. Immunoreactive bands were detected by western blot analysis with antibodies against furin, c-Src, MT1-MMP, and VEGF-C. In some experiments, the secondary antibody was substituted by Clean-Blot IP Detection Reagent for clearer IP/western blot analysis results.

### Statistical analysis

Western blots were quantified by measuring the relative density of protein bands recognized by a particular antibody using Image J software (NIH, USA). The results were expressed as mean ± standard deviation. Statistical analysis was performed with a Student’s t-test for comparison of two groups and differences with P<0.05 were considered statistically significant.

## Results

### c-Src inhibitors decrease the invasive and migratory capacity of BGC-823 cells

**Figure 1:**
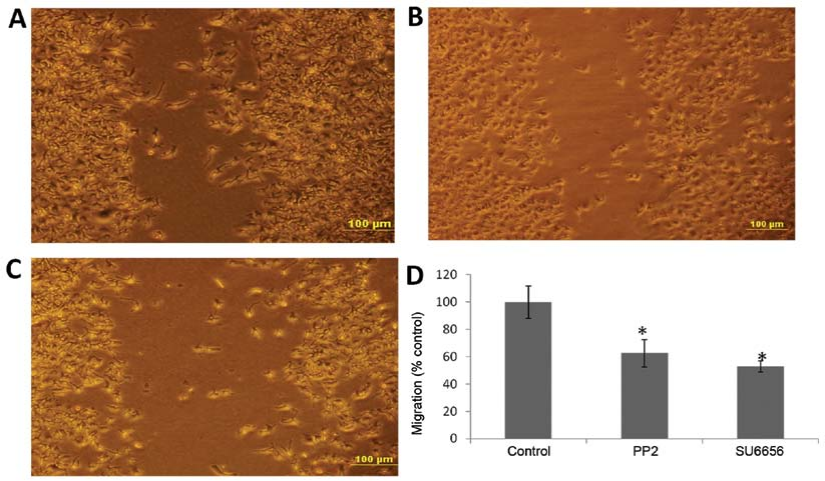
Effect of c-Src inhibitors on BGC-823 migration. BGC-823 cells (90% confluent) were wounded using a 200-μl sterile pipette tip and were then treated with c-Src inhibitors, PP2 and SU6656, for 48 h. Cell migration into the wound area was monitored by photographing the same spot with an inverted microscope equipped with a digital camera at regular intervals from 0 to 48 h. The extent of healing was defined as the ratio of the difference between the original and the remaining wound areas compared with the original wound area. (A) Control group; (B) PP2 treatment for 48 h; (C) SU6656 treatment for 48 h. (D) The graph represents the mean ± SE of at least three independent experiments. ^*^P=0.017 compared with the control.

**Figure 2:**
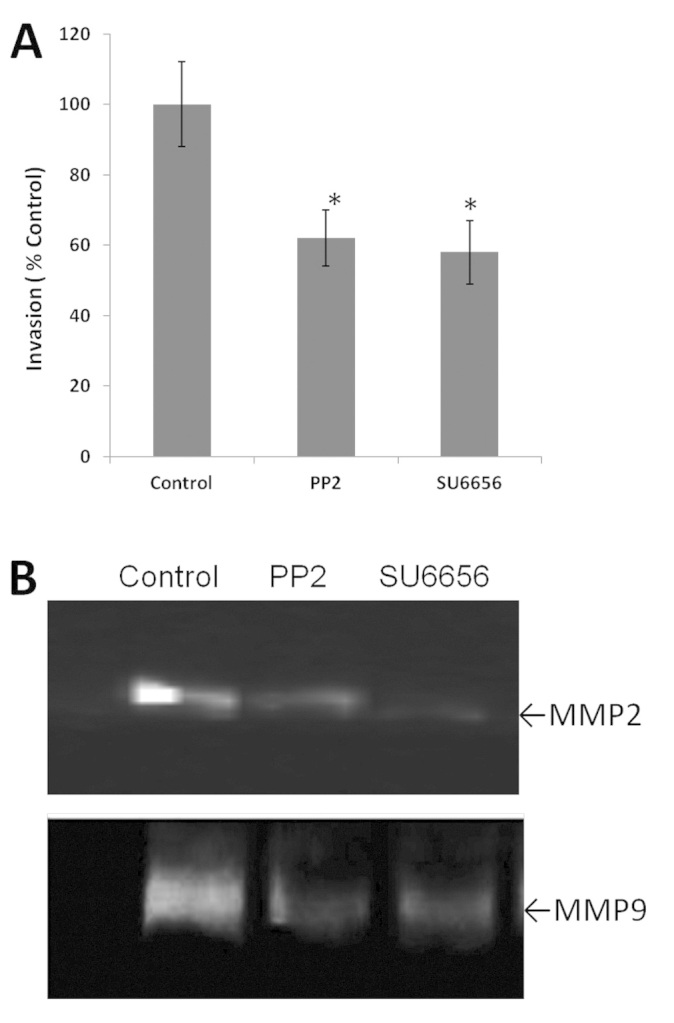
c-Src inhibitors impair the invasive ability of BGC-823 cells in correlation with downregulated MMP2 and MMP9 activity. (A) The invasive ability of BGC-823 cells treated with 10 μmol/l of either PP2 or SU6656 for 48 h was detected by the Transwell assay. Invading cells were counted for analysis. The results shown represent the mean ± SE of at least three independent experiments. ^*^P<0.05 compared with the control. (B) Identically treated cells were used to measure the activity of MMP2 and MMP9 with the zymography assay.

To detect whether the invasion and migration of BGC-823 cells were regulated by c-Src activity, we performed wound healing and Transwell assays. We observed that the invasive and migratory abilities of cells treated with either PP2 or SU6656 decreased significantly compared with those of the control (Figs. 1 and 2).

### Role of c-Src inhibitors in the activity of MMP2 and MMP9

The gelatin zymography assay was used to detect the activity of MMP2 and MMP9 after c-Src inhibitor treatment in BGC-823 cells. As shown in Fig. 2B, the activity of MMP2 and MMP9 was decreased following treatment of BGC-823 cells with either PP2 or SU6656 for 30 min prior to stimulation with PDGF-BB.

### Effects of c-Src inhibitors on the expression of furin and its substrates in BGC-823 cells

**Figure 3:**
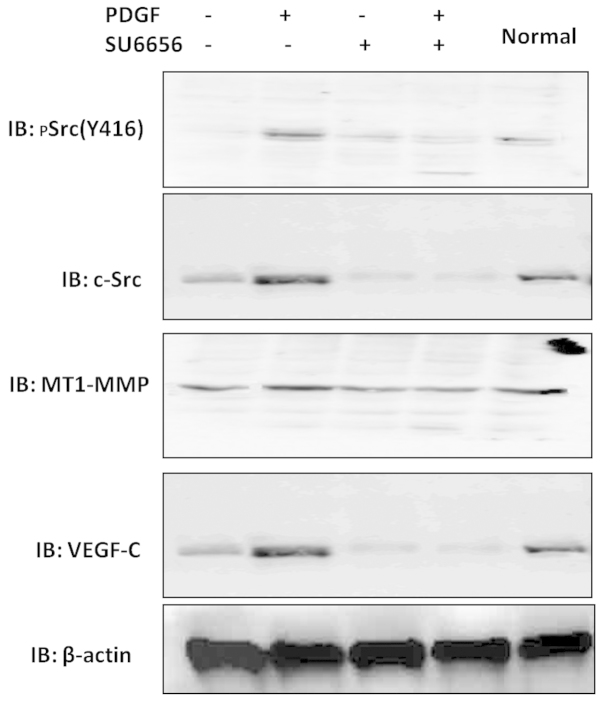
Pre-treatment of BGC-823 cells with c-Src inhibitors blocks PDGF-induced upregulation of MT1-MMP and VEGF-C. BGC-823 cells were serum starved overnight, followed by incubation with 20 ng/ml PDGF-BB for 30 min. Some groups were pre-treated with SU6656 for 30 min, as indicated. Normal represents non-serum-starved cells. Expression of p-c-Src, MT1-MMP and VEGF-C was detected with western blot analysis. Protein loading was monitored with β-actin.

The expression levels of pSrc (Y416), MT1-MMP and VEGF-C were detected by immunoblot analysis in BGC-823 cells treated with 10 μM of either PP2 or SU6656 for 24 h. Treatment with PP2 or SU6656 resulted in a quantitative decrease in the pSrc (Y416) band intensities (Fig. 3). The protein levels of MT1-MMP and VEGF-C were also significantly decreased, whereas furin protein expression showed no obvious variation (data not shown).

### c-Src activity is required for efficient association between furin and its substrates

**Figure 4:**
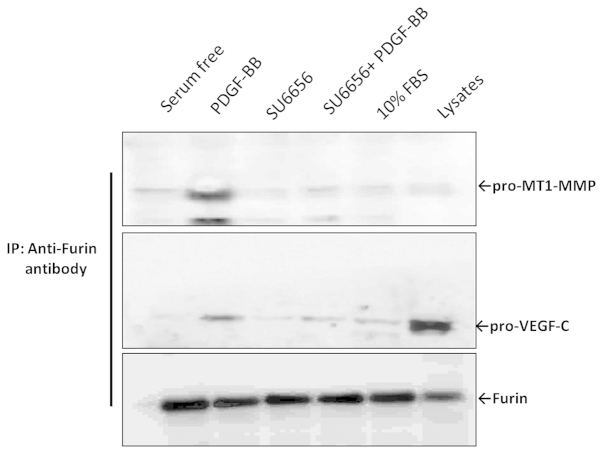
Modulation of furin interaction with its substrates by c-Src inhibition in BGC-823 cells. BGC-823 cells were serum starved overnight and were then treated with either PDGF-BB or SU6656 alone or together, as indicated. Whole cell protein lysates were collected and furin was immunoprecipitated. Furin immunoprecipitates were then examined for the expression of pre-MT1-MMP or pre-VEGF-C. Cells cultured in serum-free media, 10% FBS and total lysates were used as controls.

To explore a possible role for c-Src in the modulation of furin interaction with its substrates, we analyzed the binding between furin and two substrates, pro-MT1-MMP or pro-VEGF-C, in the presence or absence of the Src inhibitors, through co-IP experiments (Fig. 4). Briefly, BGC-823 cells were cultured in serum-free medium overnight and were then stimulated with 20 ng/ml PDGF-BB for 30 min, with or without pre-treatment with 10 μM of either PP2 or SU6656. Whole cell lysates were then collected and immunoprecipitated with the anti-furin antibody and the expression levels of MT1-MMP and VEGF-C were investigated using specific antibodies. Our results showed that MT1-MMP was found only in the PDGF-BB stimulation group, while almost no band was detected in the SU6656 pre-treated group. Similar results were observed for VEGF-C. Thus, these data suggest that c-Src activity is necessary for efficient interaction between furin and its substrates.

### c-Src directly binds to furin in vivo

**Figure 5:**
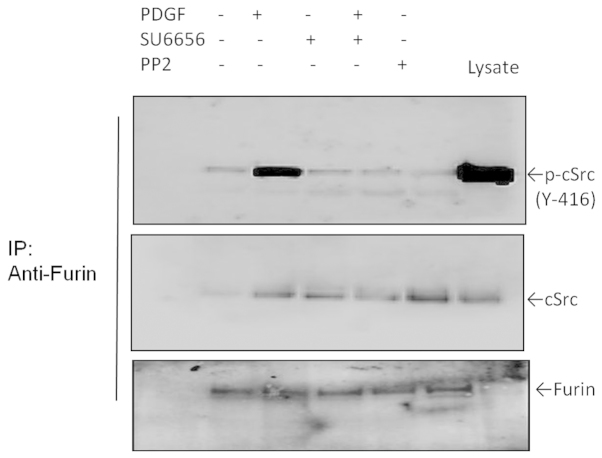
Modulation of furin interaction with c-Src by PDGF-BB and a c-Src inhibitor in BGC-823 cells. BGC-823 cells were serum starved overnight and were then treated with PDGF-BB, with and without pre-treatment with either SU6656 or PP2, as indicated. Whole cell protein lysates were collected and furin immunoprecipitation was performed. Interactions between furin and c-Src were examined by probing blots with furin or c-Src antibodies as indicated.

It is unclear whether binding between c-Src and furin exists and, if so, how it affects furin interaction with its substrates in BGC-823 cells. Thus, we performed co-IP experiments to test whether c-Src and furin are directly associated in BGC-823 cells. As shown in Fig. 5, we found that significant endogenous amounts of c-Src and furin were specifically immunoprecipitated with their respective antibodies. Notably, we found readily detectable levels of activated c-Src in the immunoprecipitations. These results suggest that endogenous c-Src may physically associate with furin *in vitro* and this binding may be required for the activity of c-Src.

## Discussion

In the present study, we demonstrated that the ability of BGC-823 cells to invade and migrate is decreased upon treatment with c-Src inhibitors. Moreover, our results indicate that c-Src activity may directly regulate BGC-823 cell invasion and migration through modulation of the maturation of MT1-MMP and VEGF-C.

Furin plays a crucial role in tumorigenesis (16,17) and it has been suggested that it could be a valuable marker for tumor progression and for predicting the outcome of this disease (18). Furin is a Ca^2+^-dependent cellular endoprotease that activates a large number of precursor proteins in secretory pathway compartments (19). Inhibition of furin activity decreases substrate activation, which has been shown to lead to both a reduced proliferation rate and invasive potential of cancer cells. Thus, furin could be a potentially useful target for anticancer therapeutics (20).

MT1-MMP and VEGF-C have been demonstrated to play vital roles in the regulation of cancer cell invasion and migration (21–23). Upregulation of MT1-MMP can effectively elevate invasiveness in human cancer cells, including gastric cancer (24–26). However, to be active, the zymogens of MT1-MMP or VEGF must be cleaved from the propeptides by the protein convertase furin (7,9,27).

Stawowy *et al* demonstrated that furin-like proprotein convertase PC5 is strongly upregulated by PDGF-BB through the PI3-kinase/p70s6-kinase pathway (28). We hypothesized that a similar mechanism may apply to the convertase furin. Thus, we first investigated whether furin or furin activity was regulated by PDGF-BB through c-Src kinase and, second, how furin activity is controlled to mediate the processing of two of its substrates, MT1-MMP and VEGF-C.

To this end, we explored the effects of c-Src inhibitors, PP2 and SU6656, on the regulation of cell migration, invasion and the protein expression of MT1-MMP and VEGF-C in BGC-823 cells. The results showed that MT1-MMP and VEGF-C protein expression levels were decreased significantly in accordance with reduced c-Src activity, while the protein level of furin remained clearly unchanged (Figs. 3 and 4). These results indicated that the regulation of MT1-MMP or VEGF-C was not dependent on the alteration of furin protein expression levels. Therefore, another mechanism should exist. Based on the above findings and accumulating evidence in the literature, we proposed that c-Src may have a potential role in the regulation of furin-mediated maturation of its substrates.

Indeed, our results showed that while activation of c-Src with PDGF-BB enhanced formation of a complex between furin and pro-MT1-MMP, SU6656 treatment resulted in the reversion of this interaction. Therefore, these data suggest that c-Src activity is required for efficient association between furin and its substrate pro-MT1-MMP. Similar results were observed when the interaction between furin and VEGF-C was examined. Notably, we found that c-Src directly interacts with furin *in vivo* in BGC-823 cells. This interaction may have a potential role in the regulation of furin-mediated maturation of its substrates.

In conclusion, our present study indicates that binding between furin and pro-MT1-MMP/pro-VEGF is enhanced upon c-Src activation. In contrast, the binding is decreased significantly following c-Src inhibitor treatment. Hence, c-Src activity may be used as a potential anticancer research approach. Therefore, the binding between furin with pro-MT1-MMP or pro-VEGF-C or other tumor-associated enzyme precursors can be regulated by c-Src activity, thereby reducing or changing the expression of these enzymes in order to inhibit the development of gastric cancer invasion and metastasis.
